# Targeting the degradation of AXL receptor tyrosine kinase to overcome resistance in gefitinib-resistant non-small cell lung cancer

**DOI:** 10.18632/oncotarget.3380

**Published:** 2015-02-26

**Authors:** Song Yi Bae, Ji-Young Hong, Hye-Jung Lee, Hyen Joo Park, Sang Kook Lee

**Affiliations:** ^1^ College of Pharmacy, Seoul National University, Seoul 151–742, Korea

**Keywords:** AXL, EGFR-TKI resistance, NSCLC, PS-RIP, yuanhuadine

## Abstract

Acquired resistance to epidermal growth factor receptor tyrosine kinase inhibitors (EGFR-TKIs), such as gefitinib, remains a major problem in non-small cell lung cancer (NSCLC) treatment. Increased activation of AXL has been identified as a novel mechanism for acquired resistance to EGFR-TKIs in NSCLC treatment. However, the cause of uncontrolled AXL expression is not fully understood. Here, we first demonstrate that AXL is overexpressed in an acquired gefitinib-resistant cell line (H292-Gef) as a result of slow turnover and that AXL is degraded by presenilin-dependent regulated intramembrane proteolysis (PS-RIP). Based on the findings, we attempted to enhance AXL degradation to overcome acquired gefitinib-resistance by the treatment of gefitinib-resistant NSCLC cells with yuanhuadine (YD), a potent antitumor agent in NSCLC. Treatment with YD effectively suppressed the cancer cell survival *in vitro* and *in vivo*. Mechanistically, YD accelerated the turnover of AXL by PS-RIP and resulted in the down-regulation of the full-length AXL. Therefore, the modulation of the proteolytic process through degradation of overexpressed AXL may be an attractive therapeutic strategy for the treatment of NSCLC and EGFR-TKI-resistant NSCLC.

## INTRODUCTION

Non-small cell lung cancer (NSCLC) is one of the leading causes of cancer-associated death, accounting for approximately 80–85% of all lung cancers [1, 2]. The occurrence of drug resistance in tumor cells has been a major barrier in anti-cancer chemotherapies. Similarly, acquired resistance has been observed in NSCLC patients treated with epidermal growth factor receptor tyrosine kinase inhibitors (EGFR-TKIs) who initially showed an excellent response to the treatment [3]. Several resistance mechanisms, such as secondary mutations, MET amplification, and activation of other receptor tyrosine kinases (RTKs) have provided a variety of different therapeutic approaches [4, 5].

Recent studies have reported that the overexpression of AXL correlates with resistance to EGFR-TKIs in NSCLC [6, 7]. AXL is a member of the TAM RTK family, which includes Tyro-3, AXL and MER. The TAM receptors are composed of two immunoglobulin-like domains and dual fibronectin type III repeats in the extracellular region, which lead to the interaction of cells with neighboring cells, and a cytoplasmic kinase domain [8, 9]. The ligand for AXL is growth-arrest-specific 6 (Gas6). AXL signaling is associated with cell survival, proliferation, invasion, metastasis, and anti-apoptosis [10, 11]. The activation of AXL may also consequently affect the downstream phosphoinositide 3-kinase (PI3K)/AKT, signal transducer and activator of transcription 3 (STAT3) and mitogen-activated protein kinase (MAPK) signaling pathways [12–14].

Down-regulation of the aberrantly activated RTK is an essential strategy for the suppression of tumor growth. The levels of some RTKs, such as MET and insulin-like growth factor 1 receptor (IGF-1R), are regulated by presenilin-dependent regulated intramembrane proteolysis (PS-RIP)-associated degradation, a proteolytic process that consists of two sequential steps [15, 16]. The first step is an ectodomain shedding, which is attributed to matrix metalloproteases (MMP) and a disintegrins and metalloproteases (ADAM), and releases the extracellular domains into the cultured media [17, 18]. The second step is an intramembrane cleavage by the γ-secretase complex, which includes anterior pharynx-defective 1, nicastrin, presenilin enhancer 2 and presenilin 1–2 (PSEN1–2). Therefore, an extracellular soluble N-terminal fragment (NTF), an intermediate membrane-anchored C-terminal fragment (CTF) and an intracellular domain (ICD) are generated by PS-RIP. ICD and NTF can contribute to the down-regulation of receptor signaling by ICD degradation, and NTF acts as a ligand decoy to inhibit the activation of the remaining membrane-anchored receptor [15].

Despite many reports on activation of AXL in drug-resistance, the cause of AXL overexpression has been poorly discussed. In this study, we present that PS-RIP is involved in the degradation of AXL, and this process may be impaired in AXL-overexpressing gefitinib-resistant NSCLC cells. Furthermore, we applied yuanhuadine (YD), an antitumor agent [19], to confirm the effect of enhancing AXL degradation on cancer cell proliferation. Thus, we suggest that targeting the proteolytic degradation of AXL is an attractive therapeutic strategy for the treatment of NSCLC and EFGR-TKI-resistant NSCLC.

## RESULTS

### AXL is up-regulated in gefitinib-resistant cell lines

**Figure 1:**
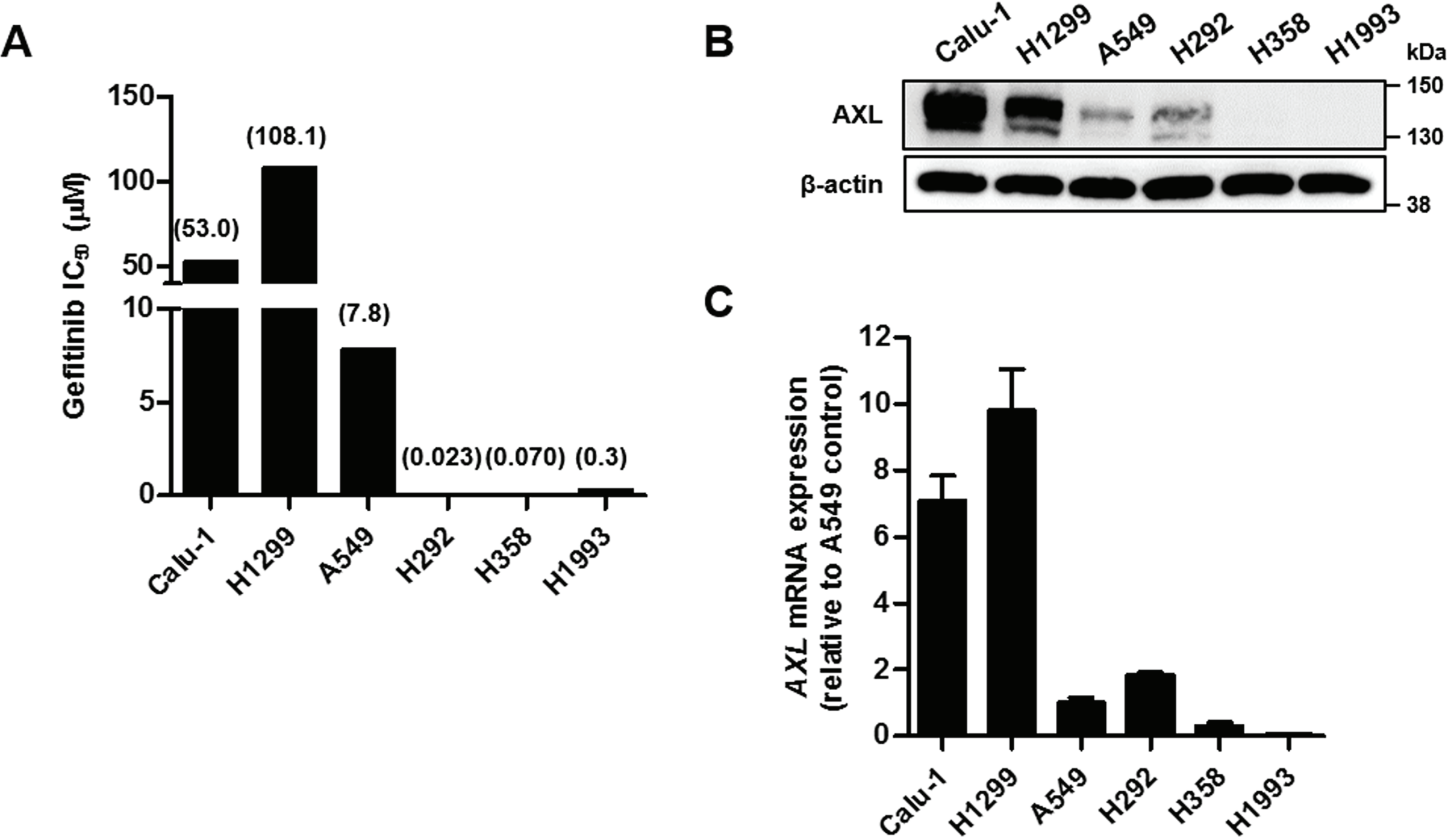
Expression of AXL in Lung Cancer Cell Lines **(A)** The cells were treated with gefitinib for 72 h, and the cell growth was then determined by SRB assay. The IC_50_ values were calculated using the TableCurve 2D software, and are shown in parentheses. **(B)** The cells were lysed, and the levels of AXL were analyzed by western blot analysis with antibody against C-terminal AXL using β-actin as a loading control. **(C)** The mRNA levels of *AXL* were examined using real-time PCR, and the *β-actin* mRNA levels were used for normalization. The data are presented as the mean fold changes ± SD relative to the A549 control. The results are representative of two (A, B) or three (C) independent experiments.

The identification of the causes of drug resistance in anti-cancer chemotherapies is important for the restoration of drug sensitivity. Recent reports suggest that the activation of AXL is in part associated with EGFR-TKI-resistant NSCLC [6, 7]. Therefore, we assessed the correlation between AXL expression and gefitinib, an EGFR-TKI, sensitivity. We first evaluated the IC_50_ values of gefitinib in six NSCLC cell lines (Figure 1A and Supplementary Figure 1). Six cell lines were treated with various concentrations of gefitinib ranging from 1.3 nM to 200 μM for 72 h. The growth inhibitory activity was determined by measuring the protein contents of cells using the sulforhodamine B (SRB) assay. Calu-1 and H1299 cells were resistant to gefitinib (IC_50_ > 10 μM), and A549 cells were regarded as intermediate-sensitive to gefitinib (IC_50_ = 7.8 μM). H292, H358 and H1993 cells were sensitive to gefitinib with the IC_50_ values of less than 1 μM. To examine whether the difference in the gefitinib sensitivity of the three groups of cell lines is related to the expression of AXL, we evaluated the AXL expression levels in these six cell lines. The AXL protein levels were considerably high in Calu-1 and H1299 cells, but the AXL protein was barely detected in the remaining four cell lines (Figure 1B). The *AXL* gene levels in the two gefitinib-resistant cells were 7- to 10-fold higher than those observed in A549 cells (Figure 1C). Although H292 cells exhibited a higher expression level of the *AXL* gene compared with A549 cells, the *AXL* gene levels in the gefitinib-sensitive cell lines were fairly low relative to those observed in the gefitinib-resistant cell lines. Therefore, it implies that there is a correlation between high AXL expression and gefitinib-resistance in NSCLC cells, whereas no correlation was found between AXL expression and gefitinib sensitivity in the gefitinib-sensitive cells.

### Degradation of AXL is suppressed in acquired gefitinib-resistant cells

**Figure 2:**
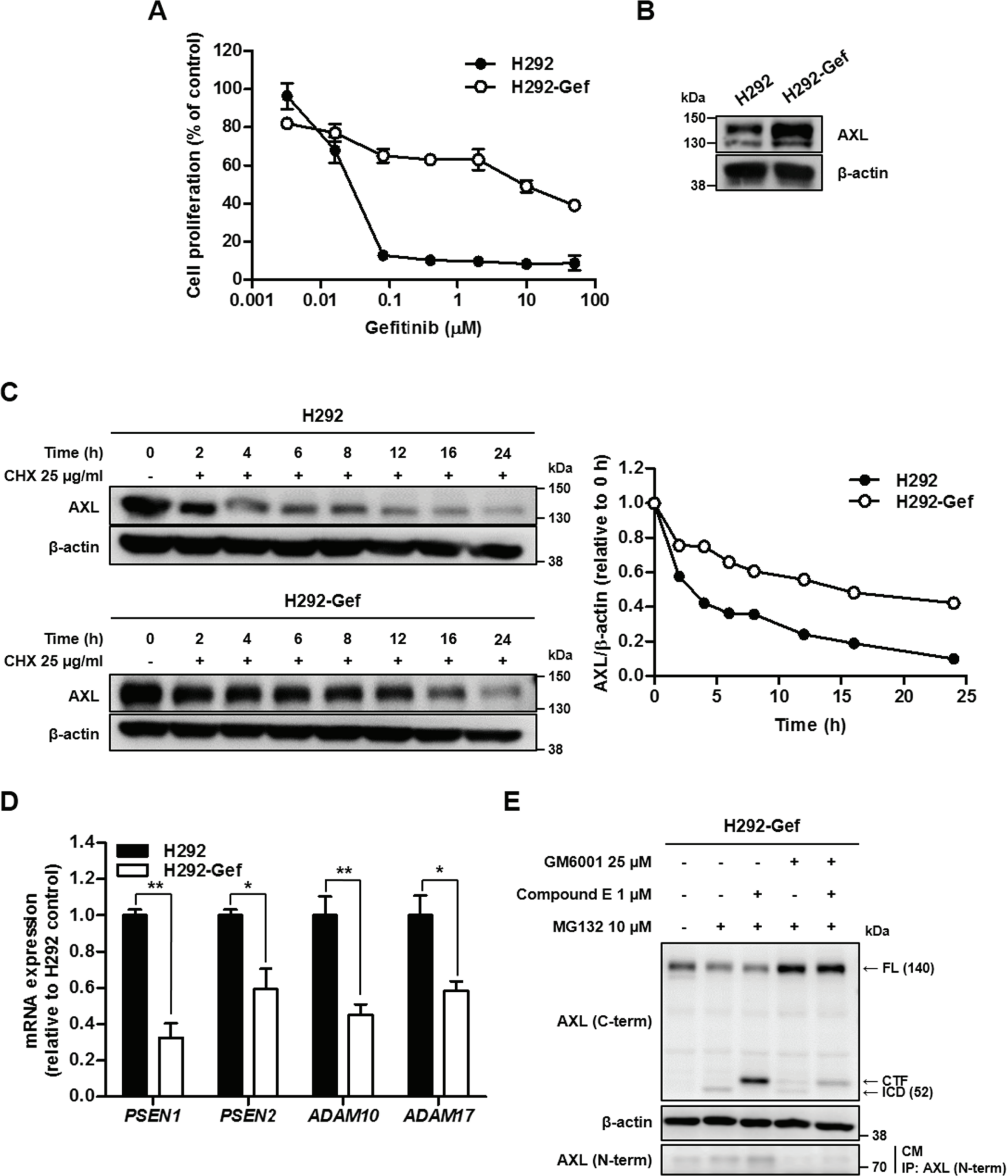
Down-regulated Turnover of AXL in Gefitinib Resistant H292 (H292-Gef) Cell Line **(A)** H292 and H292-Gef cells were treated with gefitinib for 72 h, and the proliferation of the cells was measured using the SRB assay. The IC_50_ values were calculated using the TableCurve 2D software, and the data are presented as the means ± SD. **(B)** The basal protein expression of AXL was determined by western blot using β-actin as the loading control. **(C)** The cells were treated with 25 μg/ml CHX for the indicated times. The lysates were analyzed by western blot analysis with antibody against C-terminal AXL using β-actin as a loading control. The expression levels were quantified by densitometry using ImageJ. **(D)** The mRNA expression of the indicated markers in cells was determined by real-time PCR, and the *β-actin* mRNA levels were used for normalization. The data are presented as the mean fold changes ± SD relative to the H292 control. **(E)** H292-Gef cells were treated with GM6001 and/or compound E overnight and then with MG132 for 3 h before being collected for western blot analysis using β-actin as a loading control. For determination of NTF, the culture medium (CM) was collected, immunoprecipitated with antibody against N-terminal AXL, and immunoblotted using anti-N-terminal AXL. The results are representative of two (C, E) or three (A, B, D) independent experiments. *P < 0.05, **P < 0.01, ***P < 0.005 by *t*-test.

To further investigate the status of AXL in acquired gefitinib-resistance, we established a gefitinib-resistant cell line, H292-Gef, through the continuous exposure of the parental-drug-sensitive H292 cells to gefitinib. H292-Gef cells exhibited an approximately 500-fold greater resistance to gefitinib than did the parental cells (IC_50_ value of gefitinib = 2.3 × 10^−2^ μM in H292 cells; IC_50_ value of gefitinib = 11.6 μM in H292-Gef cells, Figure 2A). Consistent with the findings in the gefitinib-resistant NSCLC cell lines, the AXL expression was markedly up-regulated in H292-Gef cells compared with H292 cells (Figure 2B). Based on the finding, we attempted to elucidate the cause of the higher AXL level in H292-Gef cells. We first determined the degradation of AXL over time by measuring AXL expression in H292 and H292-Gef cells after treatment with cycloheximide (CHX), a protein synthesis inhibitor (Figure 2C, left panel). The half-life of AXL was approximately 3 h in H292 cells and 16 h in H292-Gef cells (Figure 2C, right panel). Accordingly, we assumed that the degradation of AXL was suppressed in H292-Gef cells compared with H292 cells, and this event may be highly associated with gefitinib-acquired resistance in NSCLC cells. We then further elucidated the mechanism of AXL degradation in H292-Gef cells.

One of the mechanisms that regulate the degradation of RTK involves PS-RIP [15, 16]. Therefore, we evaluated the levels of key biomarkers in PS-RIP, namely *ADAM10*, *ADAM17*, *PSEN1* and *PSEN2*, in both H292 and H292-Gef cells. The levels of *ADAM10* and *ADAM17*, which are ADAM family members, were assessed to reflect the ectodomain shedding capacity of the cells. *PSEN1* and *PSEN2*, the catalytic subunits of γ-secretase complex, were measured as representatives of γ-secretase activity because they are known to be essential for the proteolytic activity of the enzyme. As shown in Figure 2D, all of these biomarkers were expressed at lower levels in H292-Gef cells than in H292 cells. To further verify that PS-RIP is involved in the degradation of AXL, we inhibited the two sequential steps of PS-RIP with the broad-spectrum metalloprotease inhibitor GM6001 and γ-secretase inhibitor compound E (Figure 2E). GM6001 suppressed the initial ectodomain shedding activity in H292-Gef cells; hence, the level of full-length AXL was increased and NTF in the cultured media disappeared. Moreover, the generation of an intermediate membrane-anchored CTF and its further cleavage by the γ-secretase complex to release an ICD (~52 kDa), which is stabilized by the proteasomal inhibitor MG132, was inhibited. Furthermore, the inhibition of γ-secretase by compound E also resulted in the disappearance of ICD. In contrast, the CTF remained uncleaved and accumulated in the cells, and NTF was still detected in the cultured media. Co-treatment with GM6001 and compound E increased the full-length AXL, whereas NTF and ICD were not detected. A slight level of CTF was detected, which may have formed before the cells were affected by the inhibitors. We observed similar findings after the inhibitor treatments in H292 cells (Supplementary Figure 2). Taken together, the up-regulation of AXL in H292-Gef cells may be partly associated with a decrease in the degradation rate of AXL, which appears to be regulated through PS-RIP. Thus, the degradation mechanism of AXL could be a potent target for overcoming gefitinib-acquired resistance in cancer cells.

### Antitumor agent diminishes the full-length AXL

**Figure 3:**
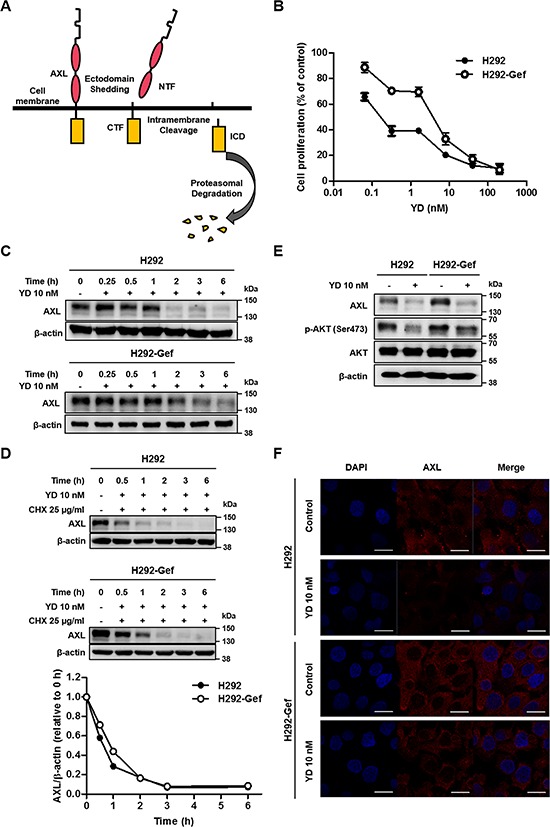
YD-induced down-regulation of the full-length AXL expression in H292 and H292-Gef Cells **(A)** Scheme of the AXL degradation by PS-RIP process. **(B)** The cells were treated with YD for 72 h, and the proliferation of the cells was determined using SRB assay. The IC_50_ values were calculated using the TableCurve 2D software, and the data are presented as the means ± SD. **(C)** The cells were treated with 10 nM YD for the indicated times, and the cell lysates were analyzed by western blot using β-actin as a loading control. **(D)** The cells were treated with 25 μg/ml CHX and 10 nM YD for the indicated times. The cell lysates were analyzed by western blot with antibody against C-terminal AXL using β-actin as a loading control. The AXL expression levels were quantified by densitometry using ImageJ. **(E)** The cells were treated with YD for 3 h, and the indicated markers were detected by western blot using β-actin as a loading control. **(F)** Cells treated with YD for 24 h were subjected to immunocytochemistry. The cells were stained with AXL and DAPI. Scale bars, 20 μm. The results are representative of three independent experiments.

We assumed that the degradation of AXL should be processed as described in Figure 3A. Overexpressed AXL could be removed from the cell membrane by increasing the cleavage of extracellular and intracellular domain of AXL. Moreover, the generated ICD could be removed through proteasomal degradation. Therefore, to confirm our hypothesis and explore the potential to inhibit cell proliferation by regulating the degradation of AXL, we applied YD, a natural product-derived antitumor agent [19, 20], to H292 and H292-Gef cells. YD effectively suppressed the proliferation of these two cell lines with IC_50_ values of 0.1 nM in H292 cells and 4.3 nM in H292-Gef cells (72 h; Figure 3B). The alterations in AXL expression was then monitored for up to 6 h after treatment with 10 nM YD (Figure 3C). The level of full-length AXL was decreased time-dependently in H292 and H292-Gef cells. The decrease of AXL level was first detected at 2 h in H292 cells and 3 h in H292-Gef cells after exposure to YD. We further determined the effect of YD on the half-life of AXL. After treatment with YD and CHX, the AXL expression was detected in H292 and H292-Gef cells (Figure 3D, upper panel). In both cell lines, the half-life of AXL was observed between 0.5 and 1 h, which was faster than the half-life measured in Figure 2C (Figure 3D, lower panel). Moreover, treatment with YD for 3 h suppressed phosphorylated AKT (Ser473), one of the key molecules of AXL downstream signaling pathway, in both H292 and H292-Gef cells (Figure 3E). Consistent with the findings shown in Figure 3C, we observed the decrease of AXL expression in the cells treated with YD for 24 h by immunocytochemistry (Figure 3F). The action of YD targeted on the expression of AXL rather than the kinase activity of AXL, as an AXL kinase activity assay confirmed that YD has no effect on the kinase activity (Supplementary Figure 3). Collectively, YD effectively decreases the full-length AXL and shortens the half-life of AXL. These events consequently inhibit the growth of cancer cells by down-regulating the AXL downstream signaling pathway.

### Antitumor agent enhances the γ-secretase-mediated generation of AXL-ICD

**Figure 4:**
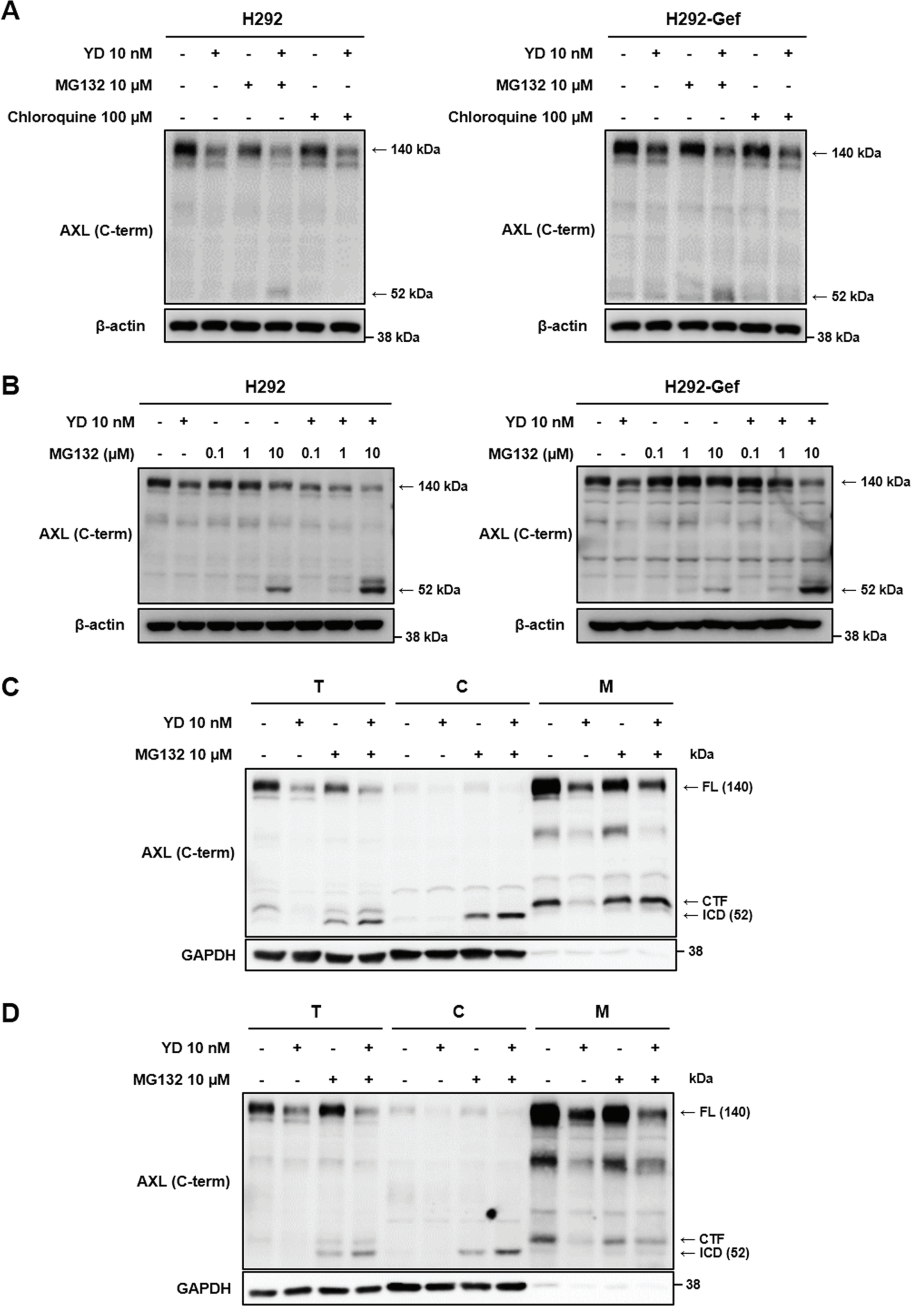
Generation of CTF and ICD of AXL by YD **(A)** The cells were treated independently with YD, MG132 (proteasomal inhibitor), and chloroquine (lysosomal inhibitor) or co-treated with YD and MG132 or with YD and chloroquine for 3 h. The cell lysates were analyzed by western blot using C-terminal AXL antibody, and β-actin served as a loading control. **(B)** The cells were co-treated with YD and the indicated concentrations of MG132. The expression of AXL fragments were detected by western blot with an antibody against C-terminal AXL using β-actin as a loading control. **(C and D)** H292 (C) and H292-Gef (D) cells were treated with YD and/or MG132 for 3 h, and the cells were then harvested and fractionated into the cytosol and membrane fractions. The lysates were subjected to western blot using anti-C-terminal AXL, and GAPDH served as a cytosol marker. T, total cell lysate; C, cytosol; M, membrane. The results are representative of two (A, B) or three (C, D) independent experiments.

We hypothesized that YD may accelerate the degradation of AXL, resulting in the reduction of full-length AXL expression. To further determine whether the proteasomal or lysosomal degradation is involved in the YD-induced decrease of AXL, we co-treated the cells with YD and the proteasomal inhibitor MG132 or the lysosomal inhibitor chloroquine (Figure 4A). Neither MG132 nor chloroquine impaired the activity of YD on AXL in both H292 and H292-Gef cells. However, the approximately 52-kDa fragment of AXL was visualized with antibody against C-terminal AXL in the cells treated with YD and MG132. In addition, to reconfirm that the generation of the 52-kDa fragment was directed by the effect of YD, the cells were co-treated with YD and the increasing doses of MG132 (Figure 4B). The 52-kDa fragment was clearly increased in the cells co-treated with YD and MG132 compared with the cells treated with MG132 alone, and a weak band was also observed above the 52-kDa fragment. However, the fragments were not visualized with the anti-AXL N-terminal antibody (Supplementary Figure 4), and this confirmed that the fragments observed consisted of the C-terminal domain of AXL. Furthermore, the cytosolic localization of the 52-kDa fragment in the cells identified that the fragment is the AXL-ICD (Figures 4C and 4D). Additionally, the fragment observed above the 52-kDa fragment was detected in the membrane fraction, indicating that the fragment corresponds to the AXL-CTF.

**Figure 5:**
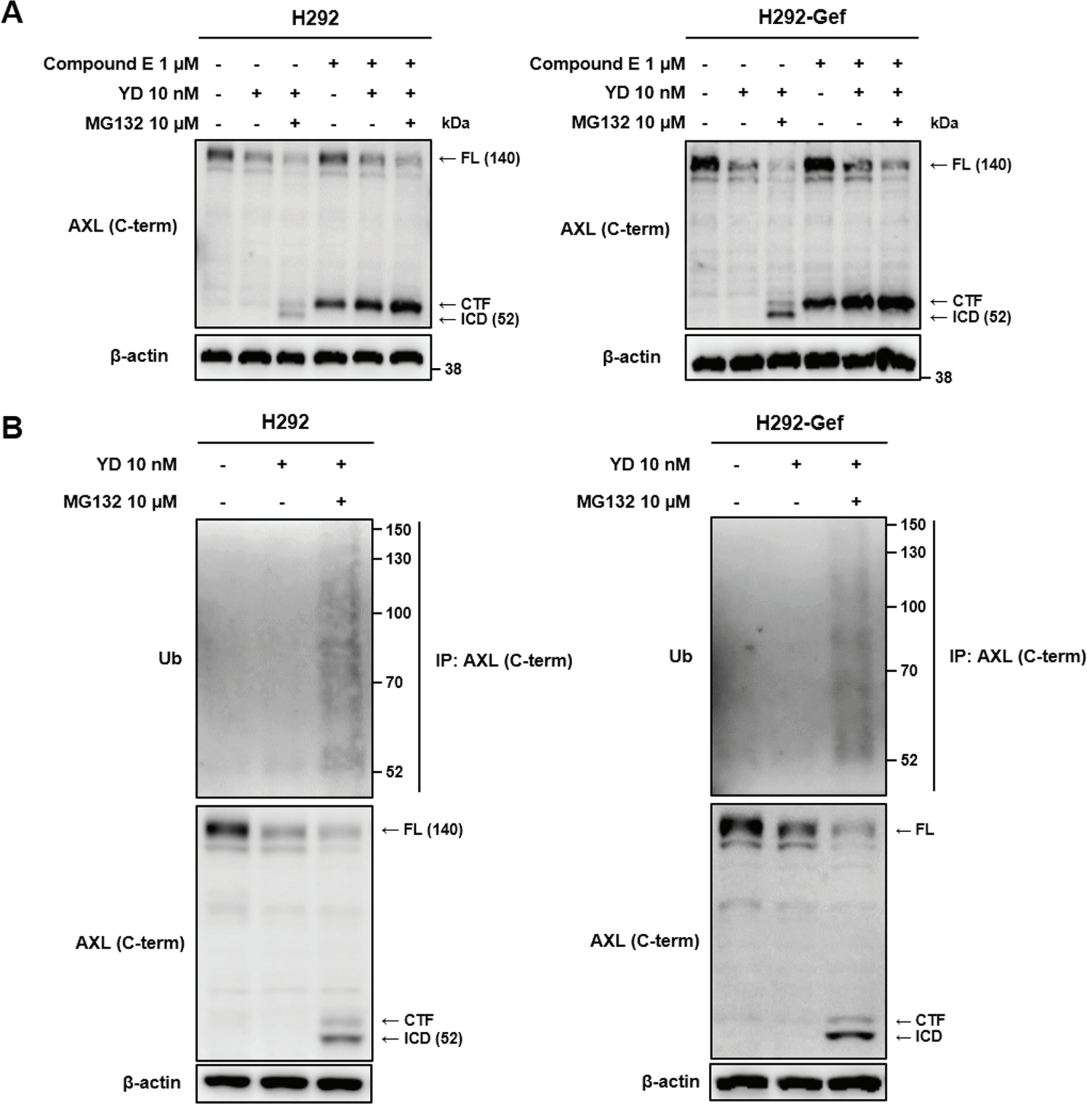
Blockage of ICD generation by γ-secretase inhibitor and fate of ICD **(A)** After overnight treatment with compound E, a γ-secretase inhibitor, the cells were treated with YD alone or co-treated with MG132 for 3 h. The collected cell lysates were analyzed by western blot with an antibody against C-terminal AXL using β-actin as a loading control. **(B)** The cells were treated with YD alone or co-treated with MG132 for 3 h. The lysates were immunoprecipitated with anti-C-terminal AXL and immunoblotted using anti-ubiquitin (Ub). AXL western blotting was performed on the total lysates. The β-actin immunoblotting of the total lysates is shown to normalize the input. The results are representative of three independent experiments.

We investigated whether γ-secretase is involved in the generation of AXL-ICD by YD. After pretreatment with compound E, the effect of YD on the release of AXL-ICD was impaired and the CTF was accumulated, whereas the full-length AXL was still decreased in both H292 and H292-Gef cells (Figure 5A). As expected, the AXL-ICD was not detected with anti-AXL N-terminal antibody (Supplementary Figure 5). We then observed the fate of the AXL-ICD produced by YD treatment. The AXL-ICD was ubiquitinated after 3 h of treatment with YD and MG132, the proteasomal inhibitor (Figure 5B). Thus, the loss of AXL induced by YD in H292 and H292-Gef cells results from the γ-secretase-mediated generation of ICD, which is rapidly removed through proteasomal degradation.

### Increased formation of AXL-NTF triggers subsequent intracellular cleavage

**Figure 6:**
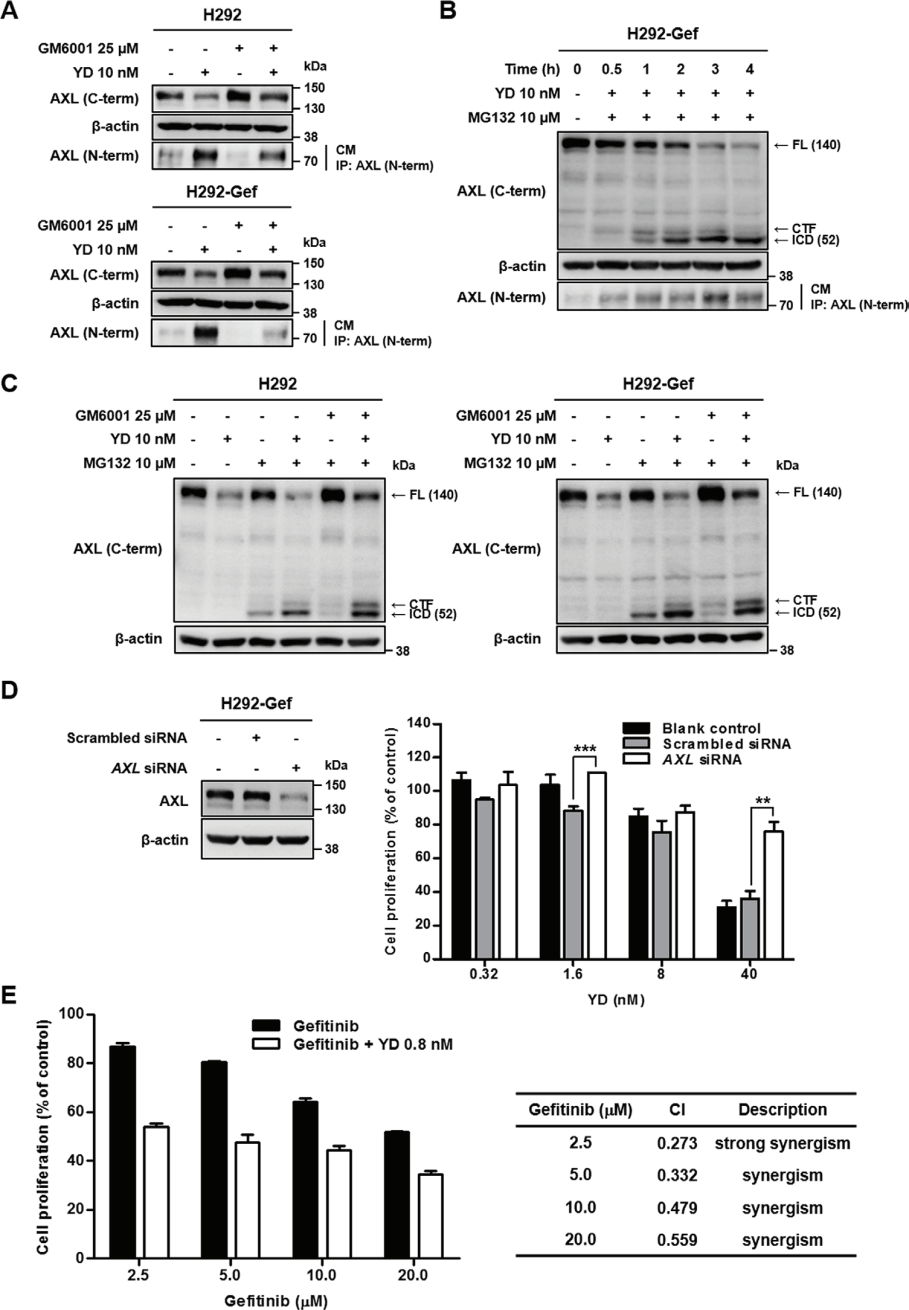
Generation of soluble AXL by YD and the combination effect of gefitinib with YD **(A)** The cells were treated or not treated with GM6001, a metalloprotease inhibitor, overnight and then treated with YD for 3 h. **(B)** H292-Gef cells were treated with YD and/or MG132 for the indicated times. The cell culture media (CM) were harvested, immunoprecipitated with antibody against N-terminal AXL, and immunoblotted using anti-N-terminal AXL. The cultured cells were lysed and analyzed by western blot with anti-C-terminal AXL using β-actin as a loading control. **(C)** The cells were pre-incubated with GM6001 overnight and then treated with YD and/or MG132 for 3 h. The lysates were subjected to western blot with antibody against C-terminal AXL using β-actin as a loading control. **(D)** H292-Gef cells were not transfected or transfected with 5 nM scrambled siRNA or *AXL* siRNA for 24 h, and the gene knockdown was confirmed by western blot with anti-C-terminal AXL using β-actin as a loading control. The transfected cells were seeded for 24 h and then treated with YD for 48 h. The cell proliferation was measured by SRB assay. **(E)** H292-Gef cells were treated with the indicated concentrations of gefitinib alone or combined with 0.8 nM YD for 48 h. The cell proliferation was determined by SRB assay and the combination effect was measured by calculating CI values. The data are presented as the means ± SD. The results are representative of two (D) or three (A, B, C, E) independent experiments. *P < 0.05, **P < 0.01, ***P < 0.005 by *t*-test.

Based on the findings that YD generates the CTF and ICD of AXL, we further investigated whether the degradation of full-length AXL by YD is associated with PS-RIP process. Without initial ectodomain removal in PS-RIP, full-length RTK is hardly cleaved by the γ-secretase complex [21]. Accordingly, we evaluated whether YD produces soluble AXL, the NTF of AXL, in addition to the CTF and ICD of AXL. After treatment of H292 and H292-Gef cells with YD for 3 h, immunoprecipitation was performed with culture media using antibody against N-terminal AXL. We detected the elevated NTF of AXL in the culture supernatant of the YD-treated groups and a reduction of full-length AXL in the cell lysates (Figure 6A). In addition, cells pre-treated with metalloprotease inhibitor GM6001 for overnight were treated with YD to determine whether the ectodomain cleavage of AXL induced by YD is metalloprotease-dependent. As shown in Figure 6A, despite the inhibition of metalloproteases, YD treatment produced AXL-NTF in the culture media. To determine the order of NTF and ICD generation, the formation of fragments in H292-Gef cells was monitored by time-course treatment with YD. MG132 was co-treated for the detection of AXL-ICD. After 0.5 h treatment with YD, AXL-NTF was observed in the culture media and only CTF was weakly detected in the cell lysates (Figure 6B). AXL-ICD appeared in the cell lysates after 1 h treatment with YD and the fragment level was increased time-dependently. To evaluate whether the interruption of metalloproteases affects the production of AXL-CTF and –ICD by YD, we incubated the cells overnight with GM6001 and then treated them with YD and/or MG132. The increase of ICD and the decrease of full-length AXL in the cells co-treated with YD and MG132 were not reversed by GM6001 pretreatment (Figure 6C). However, the cells co-treated with GM6001 and MG132 failed to release the CTF and ICD, and elevated the expression of full-length AXL. Collectively, the results indicate that YD metalloprotease-independently enhances the cleavage of the extracellular domain of AXL and efficiently provides the AXL-CTF, which acts as a substrate for the γ–secretase complex. Moreover, the cleavage of AXL induced by YD occurs through the two sequential steps of PS-RIP, the ectodomain shedding and the intramembrane cleavage.

To further explore the significance of AXL in the anti-proliferative activity of YD, H292-Gef cells were transfected with *AXL* small interfering RNA (siRNA) for 24 h, and the cell proliferation was then determined after 48 h treatment with YD. *AXL* siRNA effectively suppressed the protein expression of AXL in H292-Gef cells (Figure 6D, left panel). *AXL* siRNA-transfected cells were less sensitive to YD than were the non-transfected and scrambled siRNA-transfected cells (Figure 6D, right panel). In the presence of 40 nM YD, the cell proliferation was increased approximately 2-fold in the *AXL* siRNA-transfected cells (75.8% cell survival) compared with the scrambled siRNA-transfected cells (35.9% cell survival). We then examined whether YD-induced AXL down-regulation affects the gefitinib sensitivity in H292-Gef cells. The cells were treated with the indicated concentrations of gefitinib and YD (0.8 nM) or gefitinib alone for 48 h (Figure 6E). The combination of gefitinib and YD effectively inhibited the cell proliferation of H292-Gef cells compared with gefitinib alone. Collectively, YD appears to target the full-length AXL and thus the combination of YD and gefitinib shows a synergistic inhibitory effect on the growth of AXL overexpressing gefitinib-resistant cells.

### Antitumor agent suppresses tumor growth and AXL expression in H292 and H292-Gef cell-implanted xenografts

**Figure 7:**
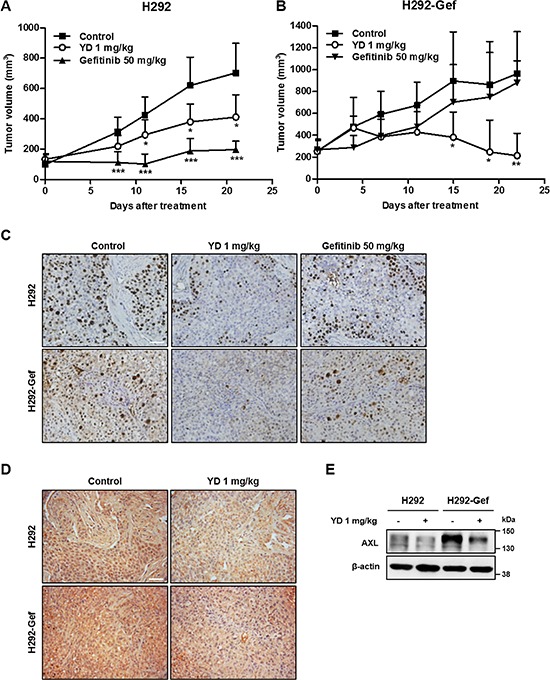
Alteration of AXL expression by YD in tumor xenograft model **(A)** H292 cells were implanted subcutaneously into the flanks of BALB/c-nude mice. Dosing of YD (1 mg/kg body weight) or gefitinib (50 mg/kg body weight) was initiated when the tumor volumes reached approximately 110 mm^3^. YD and gefitinib were administered orally once a day for 21 days. The tumor volumes were measured on Days 0 and 8 and then every five days during the remaining period (*n* = 5 mice per group). The error bars represent the means ± SD. **(B)** BALB/c-nude mice bearing H292-Gef tumors were orally treated with YD (1 mg/kg body weight) or gefitinib (50 mg/kg body weight) once a day for 22 days. The treatment was initiated when the tumor sizes reached approximately 250 mm^3^. The tumor volumes were measured every 3–4 days (*n* = 5 mice per group). The error bars represent the means ± SD. (C and D) Immunohistochemical analysis of Ki-67 **(C)** and AXL **(D)** was performed using anti-Ki-67 antibody or anti-AXL antibody directed against the C-terminal domain in tumor tissue sections. Scale bar, 50 μm. Histological images shown are representative of three independent experiments. **(E)** The expression of AXL protein in the frozen xenograft tumor tissue samples was investigated by western blot using β-actin as the loading control. Western blots are representative of three independent experiments. *P < 0.05, **P < 0.01, ***P < 0.005 by *t*-test.

We further evaluated the antitumor activity of YD in nude mouse tumor xenograft models implanted with H292 or H292-Gef cells. BALB/c-nude mice bearing xenograft tumors were orally administered 1 mg/kg YD or 50 mg/kg gefitinib once a day for 21–22 days (Figures 7A and 7B). Consistent with the *in vitro* result on the sensitivity of H292 and H292-Gef cells to gefitinib, the growth of H292 xenograft tumors was significantly suppressed by treatment with gefitinib (87% tumor growth inhibition, *P* = 0.002), but the growth of H292-Gef xenograft tumors was barely inhibited (14% tumor growth inhibition, *P* = 0.4). However, treatment with YD efficiently inhibited tumor growth of H292 xenografts by 54% (*P* = 0.01) and that of H292-Gef xenografts by 106% (*P* = 0.007, 16% tumor regression) at the end of the study. The immunohistochemical analysis of the tumor sections demonstrated the decreased expression of Ki-67, a proliferation marker, in YD-treated groups (Figure 7C). In gefitinib-treated groups, Ki-67 expression was only decreased in H292 xenograft tumors. No overt toxicity or change in body weight was observed in the treatment groups (Supplementary Figures 6A and 6B).

To validate the findings associated with AXL in tumor models, the expression of AXL in tumors obtained from the *in vivo* study was also investigated by immunohistochemistry. AXL levels were higher in the H292-Gef tumors than was the H292 tumors, and were remarkably suppressed by treatment with YD (Figure 7D). The western blot analysis also confirmed that the expression of full-length AXL was decreased in tumors of YD-treated groups (Figure 7E). Thus, the results reveal that YD effectively suppressed the growth of H292 and H292-Gef xenograft tumors as well as the expression of AXL in these tumor tissues.

## DISCUSSION

In this study, we report that the overexpression of AXL in acquired gefitinib-resistant NSCLC cells arises from the down-regulated degradation of the receptor. This finding is meaningful because although the activation of AXL has been suggested as a resistance mechanism in EGFR-TKI-resistant NSCLC cells, but the fate or turnover of the receptor has been poorly elucidated. We also demonstrate that one of the degradation mechanisms of AXL is associated with PS-RIP, and thus, enhancing the degradation of AXL can effectively inhibit cell proliferation and tumor growth in NSCLC and EGFR-TKI-resistant NSCLC cells.

High expression of AXL has been observed in various types of cancer patients and proposed as a poor prognostic biomarker [22, 23]. A large body of evidence suggested that the activation of AXL is associated with TKI-resistance in several cancers, such as gastrointestinal stromal tumors, ovarian cancer, triple-negative breast cancer and chronic myeloid leukemia [24–26]. We herein found high expression of AXL in both acquired- and non-acquired gefitinib-resistant NSCLC cells. Based on the elevated AXL expression featured in H292-Gef cells, we proceeded to elucidate the mechanism underlying how AXL is overexpressed in drug-resistant cells. Primarily, we found that the half-life of AXL protein was increased in H292-Gef cells compared with the parent H292 cells. This event suggests that the sustained higher level of AXL protein in the acquired-gefitinib-resistant cells may be associated with down-regulation of the degradation of AXL in the cells. The levels of many RTKs are regulated by a proteolytic cleavage process, such as PS-RIP [27]. AXL has been reported to undergo post-translational processing by an unknown protease to release NTF and CTF, an event that is augmented by phorbol ester treatment [28]. We identified the constitutive release of the AXL-NTF, CTF and ICD in H292 and H292-Gef cells. We also found that inhibition of the two sequential steps of PS-RIP interrupted the formation of the three fragments of AXL, indicating that PS-RIP plays a role in AXL regulation. Furthermore, the key markers of PS-RIP, such as *ADAM10*, *ADAM17*, *PSEN1*, and *PSEN2*, are down-regulated in H292-Gef cells. Thus, the mechanism of AXL overexpression in acquired-gefitinib-resistant cells may be dysregulation of PS-RIP, resulting in an elevation in the half-life of the AXL receptor.

Accordingly, we speculated that the activation of PS-RIP may be a potent therapeutic strategy for regulating abnormal activation of AXL in NSCLC cells. To validate this assumption, we applied YD, an antitumor agent isolated from flowers of *Daphne genkwa* that effectively inhibits the growth of gefitinib-resistant NSCLC cells and that of A549 NSCLC xenograft tumors [19]. YD accelerated the degradation rate of full-length AXL and shortened the half-life of AXL compared with its original half-life in H292 and H292-Gef cells. The sequential generation of AXL-NTF and –ICD was induced simultaneously with the decrease of full-length AXL by YD. The inhibition of γ–secretase activity resulted in the suppression of ICD formation and the accumulation of CTF, indicating that YD suppresses AXL through γ–secretase activity. Treatment with YD increased the release of NTF into the culture medium, and co-treatment with metalloprotease inhibitor failed to impair the down-regulation of full-length AXL and the generation of both NTF and ICD by YD. Therefore, metalloprotease-independent induction of the initial step of PS-RIP, an ectodomain shedding, by YD provides more substrates for the second step, γ-secretase-associated cleavage. Furthermore, *AXL* siRNA-transfected H292-Gef cells were less sensitive to YD treatment, providing additional evidence clarifying that YD affects the full-length AXL protein. Although, YD did not inhibit the kinase activity of AXL, the loss of AXL by YD efficiently suppressed one of the key molecules of AXL downstream signaling, such as AKT. In addition, the combination treatment with gefitinib and YD synergistically inhibited the proliferation of H292-Gef cells *in vitro*. YD also effectively suppressed the growth of H292-Gef xenograft tumors and enhanced the degradation of AXL in YD-treated xenograft tissues.

Because most ICDs generated from RTKs are labile, the fragments are degraded in a proteasome-dependent manner and consequently, the removal of kinase domain results in the down-regulation of receptor signaling [29]. Nevertheless, some ICDs act as active fragments and regulate gene transcription or the cellular survival/apoptosis balance [30, 31]. In this study, however, the AXL-ICD generated by YD is proteasome-dependently degraded. Therefore, the produced ICD is rapidly cleared rather than involved in regulating biological processes. The NTF released by PS-RIP is known to function as a ligand decoy, which would prevent the ligand from binding to RTK for signal transduction [18, 32]. The NTF generated by YD treatment may also have the potential to act as a ligand decoy for AXL. Although further studies are needed to determine the precise cleavage sites on AXL and the mechanism in which YD enhances the action of PS-RIP, the findings suggest that the modulation of AXL turnover may be a promising therapeutic target for EGFR-TKI-resistant NSCLC cells.

In summary, we suggest that the overexpression of AXL protein in an acquired-gefitinib-resistant cells is caused by the down-regulation of its turnover. We also demonstrated that PS-RIP is involved in the degradation of AXL. Therefore, these findings present the targeting of the AXL degradation mechanism as a novel therapeutic strategy in EGFR-TKI-resistant NSCLC treatment.

## MATERIALS AND METHODS

### Cell lines, reagents, antibodies and animals

The human lung carcinoma H1299, A549 and H358 cells were provided by the Korean Cell Line Bank (Seoul, Korea). The human lung carcinoma Calu-1, H292 and H1993 cells were obtained from the American Type Culture Collection (Manassas, VA, USA). The cell lines were cultured in RPMI 1640 medium supplemented with 10% FBS and antibiotics-antimycotics (PSF; 100 units/mL penicillin G sodium, 100 μg/mL streptomycin, and 250 ng/mL amphotericin B). The cells were incubated at 37°C and 5% CO_2_ in a humidified atmosphere. Gefitinib-resistant H292 cells (H292-Gef) were developed by our group from the parental H292 cells through continuous exposure to gradually increasing concentrations of gefitinib (Selleckchem. Houston, TX, USA) and maintained in RPMI 1640 medium containing 1 μM gefitinib. MG132, cycloheximide and compound E were purchased from A.G. Scientific (San Diego, CA, USA). GM6001 was obtained from Calbiochem (San Diego, CA, USA). Yuanhuadine (YD; purity > 98.5%) was isolated and identified from a CHCl_3_-soluble fraction of the flowers of *Daphne genkwa*, as described previously [19, 20]. Antibodies against C-terminal AXL, ubiquitin and β-actin were obtained from Santa Cruz Biotechnology (Santa Cruz, CA, USA). N-terminal AXL were purchased from R&D Systems (Abingdon, UK), and p-AKT (Ser473), AKT and GAPDH were obtained from Cell Signaling Technology (Danvers, MA, USA). Female nude mice (4.5 weeks of age, BALB/c-nu) were purchased from Central Laboratory Animal, Inc. (Seoul, Korea) and housed in the animal care facility at Ewha Womans University under pathogen-free conditions.

### Cell proliferation assay

The cells (5–8 × 10^4^ cells/ml) were treated with various concentrations of compounds in 96-well culture plates. After 72 h of treatment, the cells were fixed with 10% TCA solution, and the cell proliferation was determined through a sulforhodamine B (SRB) assay [33]. The percentage of cell proliferation was determined according to the following formula: cell proliferation (%) = 100 × [(A _treated_ – A _zero day_)/(A _control_ – A _zero day_)], where A is the average absorbance. The IC_50_ values were calculated through non-linear regression analysis using TableCurve 2D v5.01 (Systat Software Inc., San Jose, CA, USA)

### Western blot and immunoprecipitation analysis

For western blot analysis, the cells were lysed through boiling in 2× sample loading buffer (250 mM Tris-HCl pH 6.8, 4% SDS, 10% glycerol, 0.006% bromophenol blue, 2% β-mercaptoethanol, 50 mM sodium fluoride, and 5 mM sodium orthovanadate) and further incubated for 5 min at 100°C. Equal amounts (10 or 50 μg) of protein were subjected to 8–10% SDS-PAGE and transferred onto PVDF membranes (Millipore, Bedford, MA, USA). The blots were blocked with 5% bovine serum albumin (BSA) in Tris-buffered saline containing 0.1% Tween-20 (TBST) for 1 h at room temperature, and then incubated with primary antibodies in 2.5% BSA in TBST overnight at 4°C. The membranes were washed three times with TBST, and incubated with the corresponding secondary antibodies diluted in 2.5% BSA in TBST for 2 h at room temperature. After washing three times with TBST, the membranes were exposed to enhanced chemiluminescence (ECL) solution (Intron, Daejon, Korea). The blots were detected with an LAS-4000 (Fuji Film Corp., Tokyo, Japan).

For immunoprecipitation of the cultured medium, the cell supernatant was filtered through a 0.2-μm filter, and the protease and phosphatase inhibitors (Roche Applied Science, Penzberg, Germany) were then added. For immunoprecipitation of the cultured cells, the cells were lysed in IP lysis buffer (50 mM Tris-HCl pH 7.4, 150 mM NaCl, 1 mM EDTA, and 0.5% NP-40) containing protease and phosphatase inhibitors. The filtered cultured medium or equal amount of cell lysates was precleared for 30 min at 4°C with protein G Sepharose 4FastFlow (GE Healthcare, Little Chalfront, UK). After removal of the beads (10 min, 12 000 rpm, 4°C), the supernatant was incubated with the indicated antibody overnight at 4°C. The immunocomplex was collected with beads at 4°C for 2 h. The beads were washed four times with IP lysis buffer, and the bound proteins were eluted with 2× Laemmli sample buffer at 95°C for 10 min. The collected protein samples were subjected to western blot analysis.

### Real-time polymerase chain reaction (PCR)

The total RNA from the cells were extracted with TRI reagent (Invitrogen, Grand Island, NY, USA), and 1 μg of total RNA was reverse-transcribed using a Reverse Transcription System (Promega, Madison, MA, USA) according to the manufacturer's instructions. Real-time PCR was conducted using iQ^TM^ SYBR^®^ Green Supermix (Bio-Rad, Hercules, CA, USA), according to the manufacturer's instructions. The conditions for the assay were the following: 20 sec at 95°C, 40 cycles of 20 sec at 95°C, 20 sec at 56°C, and 30 sec at 72°C, 1 min at 95°C, and 1 min at 55°C. All of the experiments were performed in triplicate, and the analysis was performed through the comparative C_T_ method using *β-actin* for normalization. The sequences of the primers are listed in the Supplementary Table 1.

### Immunocytochemistry

The cells were grown on cover slips in dishes. After treatment, the cells were fixed with 4% paraformaldehyde (in PBS) for 15 min. The fixed cells were blocked in permeabilizing conditions with 1% BSA (in PBS) containing 0.1% Triton X-100 for 30 min at room temperature. The cells were incubated with primary antibody at 4°C overnight. Following the overnight incubation, the cells were incubated with donkey anti-goat secondary antibody conjugated to Alexa 594 (Invitrogen) for 2 h at room temperature. DAPI (0.5 μg/ml) was used to counterstain the nuclei. The images were obtained using a Zeiss ApoTome microscope (Carl Zeiss, Jena, Germany).

### Cell fractionation

The cells were washed with ice-cold PBS three times and then washed once rapidly with cold fractionation lysis buffer (20 mM Tris-Cl pH 7.4, 100 mM NaCl, 2 mM MgOAc, 5 mM KCl, 10 μM GTP, and protease inhibitor). The scraped cells in lysis buffer were incubated on ice for 10 min and then lysed by passing through a 27-gauge needle 35 times on ice. Small amounts of the total lysate samples were reserved from the lysates, and the remaining samples were centrifuged (20 min, 2 000 rpm, 4°C). The supernatant was then collected and centrifuged (30 min, 14 000 rpm, 4°C). After the second centrifugation, the resulting supernatant was kept for analysis of the cytosolic fraction, and the pellet was washed with lysis buffer for membrane fraction extraction. The lysis buffer was removed by centrifugation (20 min, 14 000 rpm, 4°C), and the membrane pellet was once again washed with ice-cold PBS. After removal of PBS (5 min, 14 000 rpm, 4°C), the membrane pellet was suspended in 1% NP-40 lysis buffer for 1 h on ice with mixing every 10 min. The total, cytosolic and membrane lysates were boiled with 2× Laemmli sample buffer at 95°C for 10 min.

### RNA interference

RNA interference of *AXL* was performed using 25-bp siRNA duplexes purchased from Invitrogen. The coding strand for *AXL* was as follows: sense CCA GCA CCU GUG GUC AUC UUA CCU U and antisense AAG GUA AGA UGA CCA CAG GUG CUG G. The cells were transfected with 5 nM siRNA duplexes using Lipofectamine RNAiMAX (Invitrogen) according to the manufacturer's instructions. Cells transfected with a control nonspecific siRNA duplex (Invitrogen) were used as controls for direct comparison.

### Analysis of drug combination

The cells were plated in 96-well culture plates and then exposed to various concentrations of gefitinib and YD (0.8 nM) in a ratio of 1:1. After 48 h incubation, the cell proliferation was determined using the SRB assay. The combination effect was evaluated by calculating the combination index (CI) values as follows: CI = D_1_/(D_*x*_)_1_ + D_2_/(D_*x*_)_2_, where D_1_ and D_2_ are the concentrations of the combined test compounds that achieve the expected effect, and (D_*x*_)_1_ and (D_*x*_)_2_ are the concentrations that achieve similar effects when the test compounds are used alone. In this study, 50% inhibition was taken as the effective level. The CI values were compared to the reference values reported by Chou [34].

### *In vivo* tumor xenograft model

All animal use and care protocols followed the guidelines of the Institutional Animal Care and Use Committee at Ewha Womans University and were approved by the Korean Association of Laboratory Animal Care. H292 or H292-Gef cells were injected subcutaneously into the flanks of the mice (1 × 10^7^ cells in 200 μl of medium). When the tumor volume reached approximately 110 mm^3^ (H292) and 250 mm^3^ (H292-Gef), the mice were randomized into the vehicle control and treatment groups (*n* = 5). YD (1 mg/kg) or gefitinib (50 mg/kg) dissolved in a volume of 200 μl of vehicle solution (Tween 80-ethanol-H_2_O, 1:1:98) was administered orally once a day for 21 days (H292) and 22 days (H292-Gef). The control group was treated with an equal volume of vehicle. The tumor size was measured using a digital slide caliper and volumes (mm^3^) were determined as follows: (width) × (length) × (height) × π/6. The percentage of tumor growth inhibition was estimated as follows: 100 × [1 – (TV_f, treated_ – TV_i, treated_)/(TV_f, control_ – TV_i, control_)], where TV_f_ is the average tumor volume at the end of the study, and TV_i_ is the average tumor volume at Day 0, the day before first administration. The percentage of tumor regression was estimated as follows: 100 × [1 – (TV_f, treated_/TV_i, treated_)]. The body weight of each mouse was also monitored for toxicity.

### Immunohistochemistry and biochemical analysis of tumors

The excised tumors were fixed in 4% paraformaldehyde (PFA) and embedded in paraffin. Sectioned slides of the embedded specimens were serially deparaffinized, rehydrated, and subjected to antigen retrieval. The slides were incubated with antibody against Ki-67 (Dako, Glostrup, Denmark) or C-terminal AXL, detected using the LSAB^TM^+ System-HRP kit (Dako) and counterstained with hematoxylin. The stained sections were observed and photographed with an inverted phase-contrast microscope. For biochemical analysis of the tumors, a portion of the frozen tumors was thawed on ice and homogenized using a hand-held homogenizer in Complete Lysis Buffer (Active Motif, Carlsbad, CA, USA). The tumor lysates were subjected to protein concentration determination assays, and the aliquots were stored at –80°C. Prior to western blot analysis, the lysates were boiled with 2× Laemmli sample buffer at 95°C for 10 min.

### Statistical analysis

The data are presented as the means ± SD for the indicated number of independently performed experiments. The statistical significance (*p* < 0.05) was assessed using Student's *t*-test. All statistical tests were two-sided.

## SUPPLEMENTARY METHODS


