# Supplementary material for: Targeting the degradation of AXL receptor tyrosine kinase to overcome resistance in gefitinib-resistant non-small cell lung cancer
Source: Oncotarget. 2015 Feb 26;6(12):10146–60. doi: 10.18632/oncotarget.3380 (PMC4496346; doi:10.18632/oncotarget.3380)
Supplement: Supplementary file 1 [file oncotarget-06-10146-s001.pdf]

SUPPLEMENTARY METHODS

Kinase activity assay

The AXL kinase activity was assayed through the ADP-Glo Kinase Assay (Promega), according to the manufacturer's instructions. Ten microliters of AXL kinase solution (36 ng, SignalChem, Richmond, Canada) were preincubated with 5 μl of 5× test compound or 5% DMSO in reaction buffer for 10 min at 30°C. The reaction was started by the addition of 10 μl of 2.5× ATP/AXLtide, the substrate for AXL kinase (SignalChem). After 1 h of incubation at 30°C, the reaction was stopped and further processed as described by the manufacturer. The kinase activity was determined by measuring the luminescence. The relative kinase activity was calculated relative to 100% kinase activity after subtraction of the negative control signal from all of the sample signals and subsequent elimination of the 0% kinase activity. BMS777607, an AXL inhibitor, was used as a reference compound.

SUPPLEMENTARY FIGURES AND TABLE

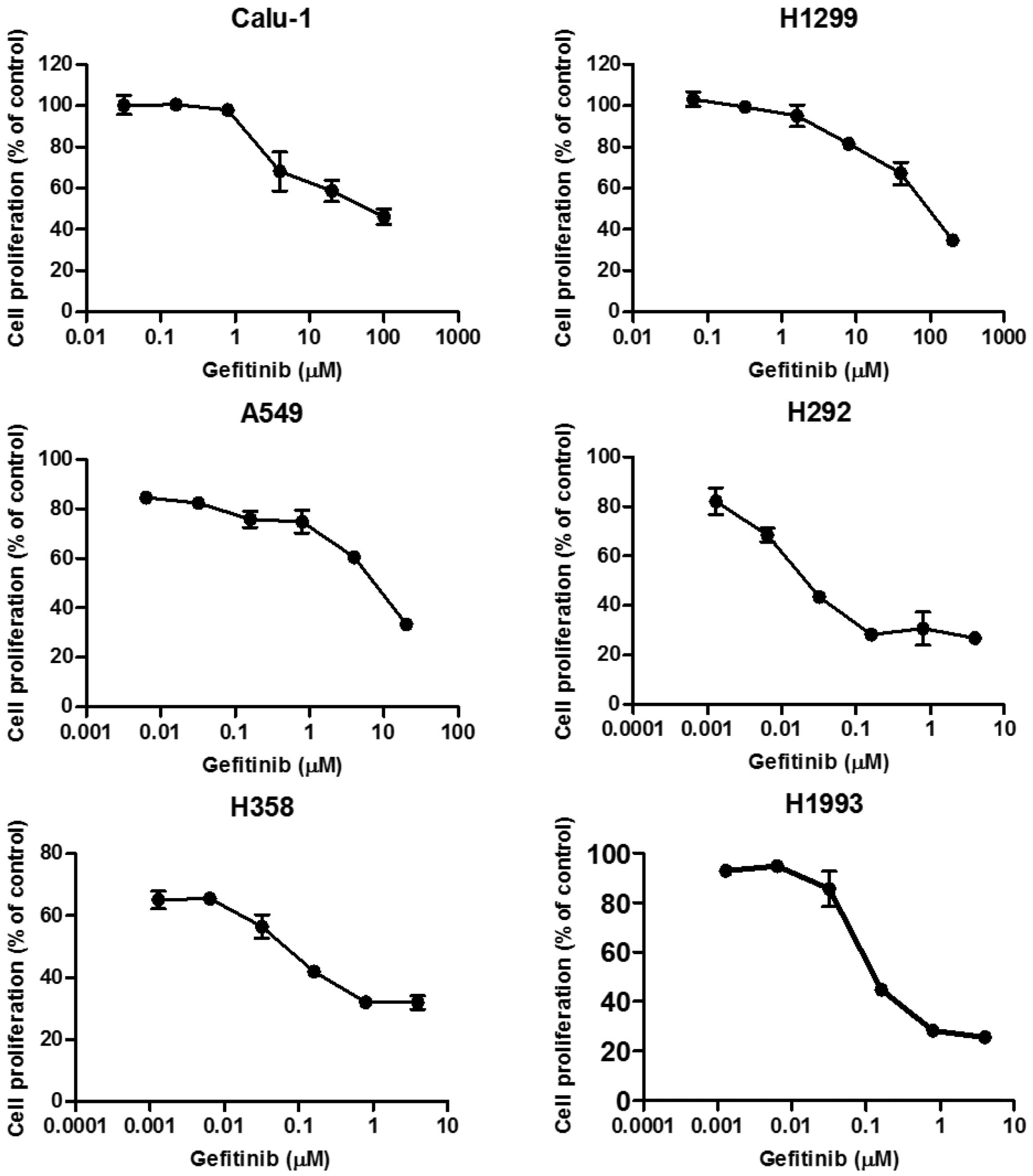

Supplementary Figure 1: Anti-proliferative effect of gefitinib on human lung cancer cell lines. The cells were treated with the indicated concentrations of gefitinib in triplicate for 72 h, and the cell growth was then determined by SRB assay. The data are presented as the means ± SD. The results are representative of two independent experiments.

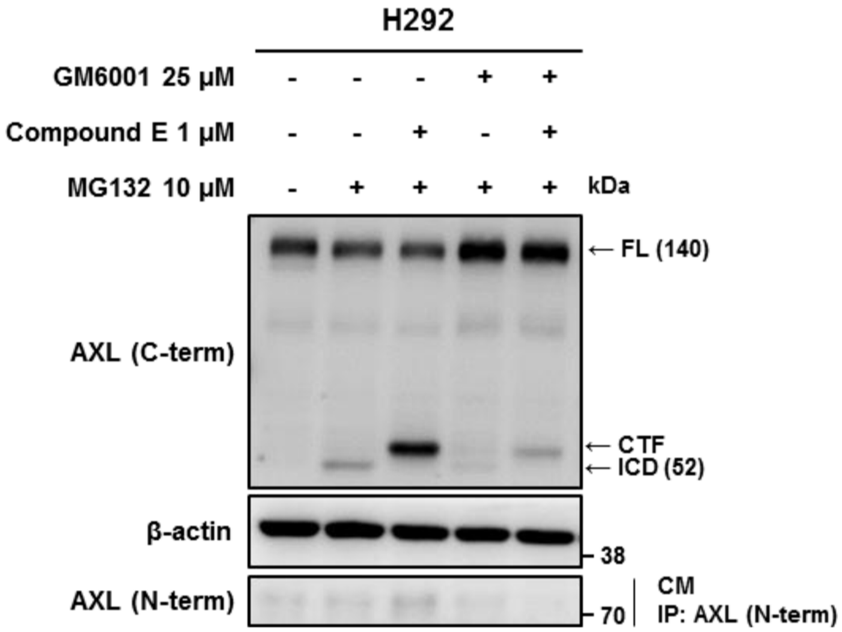

Supplementary Figure 2: PS-RIP-associated degradation of AXL in H292 cells. The cells were treated with GM6001 and/or compound E overnight and then treated with MG132 for 3 h before being collected for western blot analysis using β-actin as the loading control. For determination of NTF, the culture medium (CM) was collected, immunoprecipitated with antibody against N-terminal AXL, and immunoblotted using anti-N-terminal AXL. Representative western blots are shown.

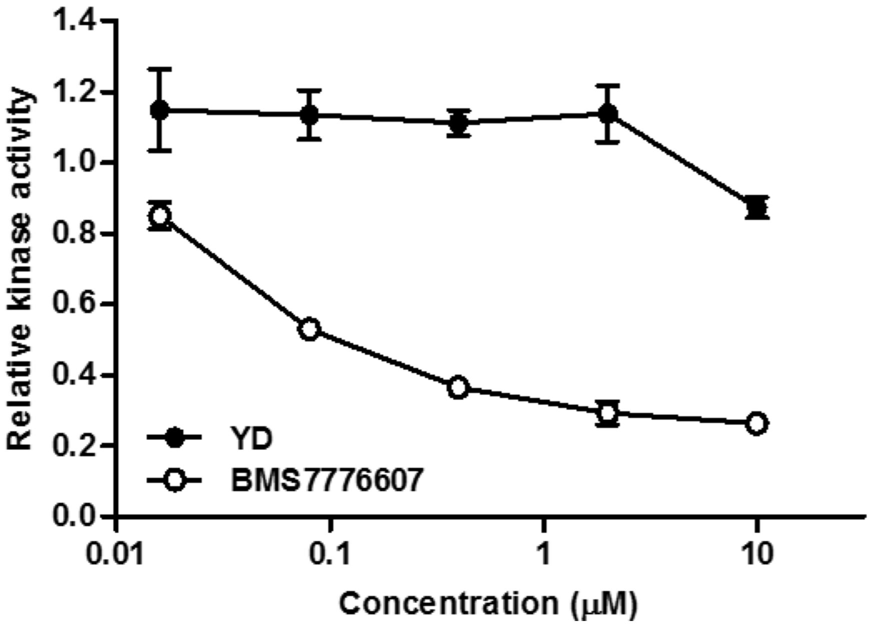

Supplementary Figure 3: Effect of YD on AXL kinase activity. YD or BMS777607 was preincubated with active AXL kinase for 10 min, and the reaction was then started by the addition of ATP and AXLtide substrate. After incubation for 1 h at 30°C, the kinase activity was determined by measuring the luminescence using the ADP-Glo Kinase Assay (Promega). BMS777607, an AXL inhibitor, was used as a reference compound. The data are presented as the means ± SD. The result is representative of two independent experiments. **P* < 0.05, ***P* < 0.01, ****P* < 0.005 by *t*-test.

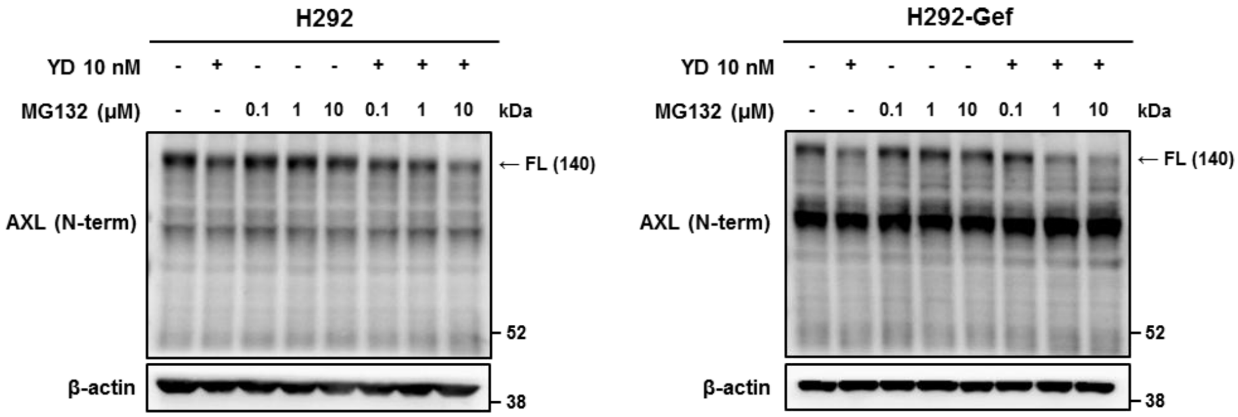

Supplementary Figure 4: Characterization of AXL fragments in H292 and H292-Gef cells. The generated fragments of AXL by YD contain the C-terminal domain. H292 and H292-Gef cells were treated with YD and/or MG132 for 3 h, and the cells were then harvested and subjected to western blot with antibody against N-terminal AXL using β-actin as the loading control. The results are representative of two independent experiments.

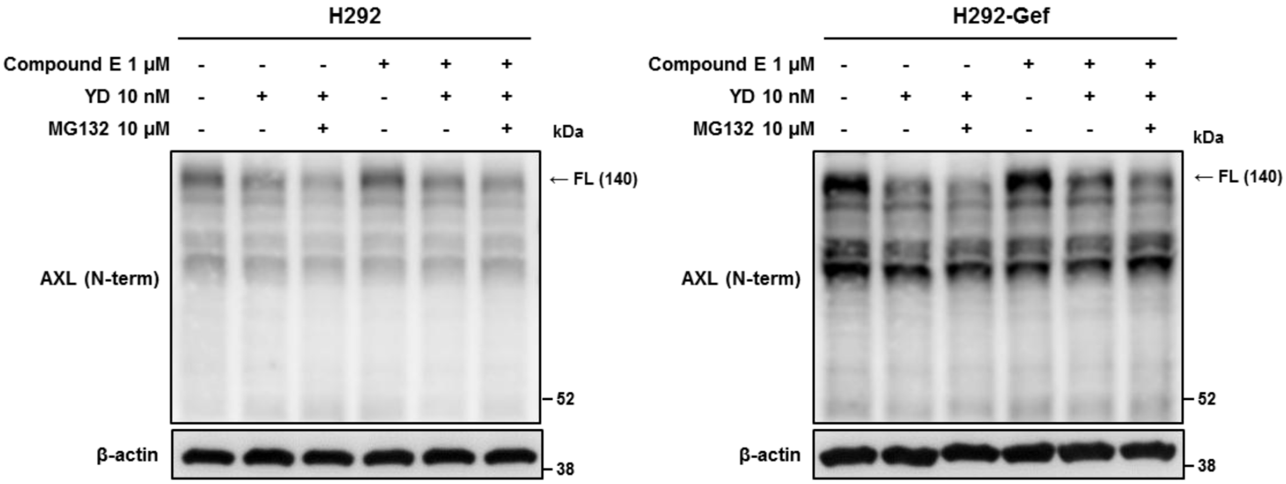

Supplementary Figure 5: Blockage of ICD generation by γ-secretase inhibitor. After overnight treatment with compound E, the cells were treated with YD and MG132 for 3 h, and the expression of AXL was then examined by western blot with an antibody against N-terminal AXL using β-actin as a loading control. Representative western blots are shown.

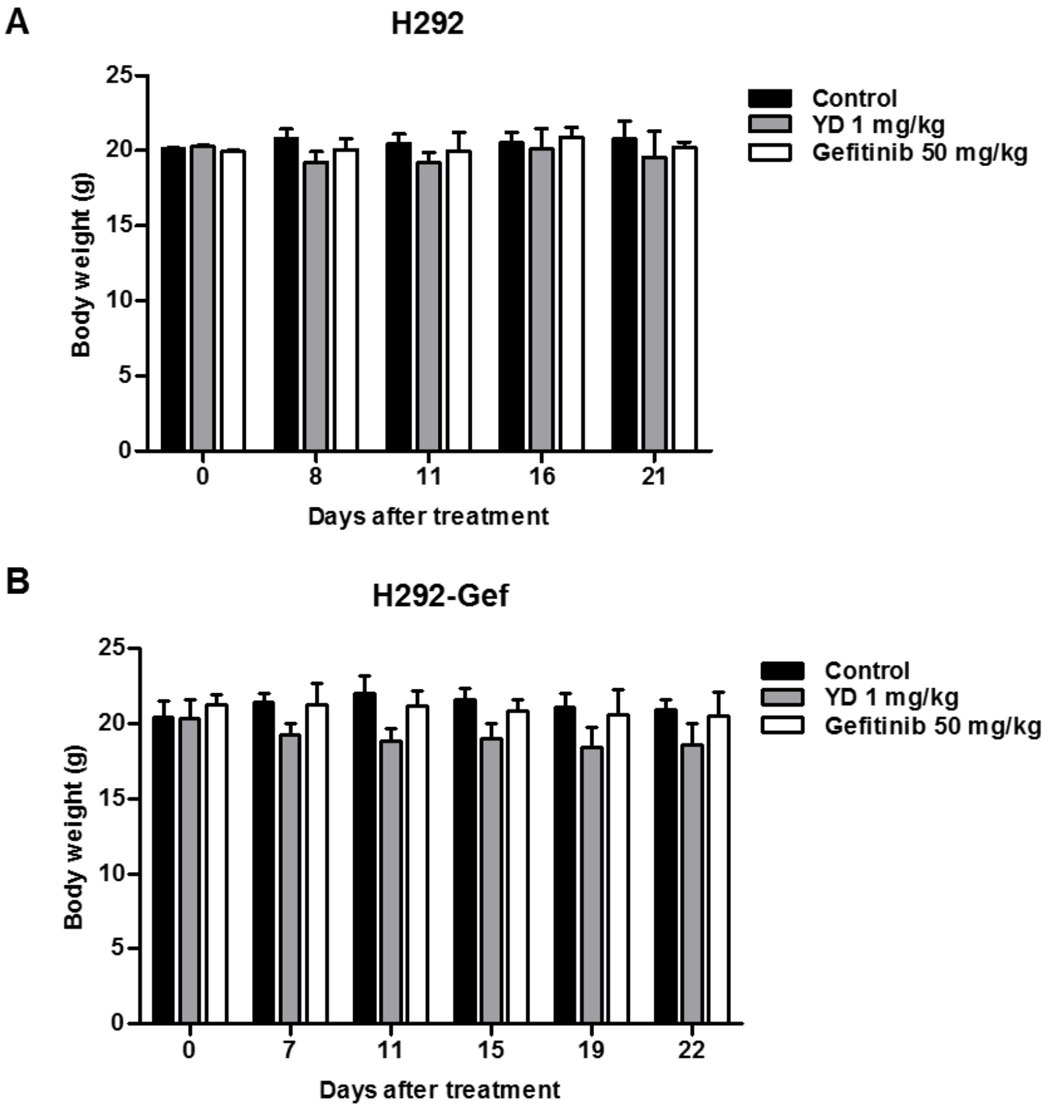

Supplementary Figure 6: Body weight measurement of the xenograft models during treatment with YD (1 mg/kg body weight) or gefitinib (50 mg/kg body weight). (A) The body weight of mice bearing H292 xenografts was determined on Day 0, the initial day of administration, Day 8, and once every three or five days during the remaining period of treatment (*n* = 5 per group). The error bars represent the means ± SD. **(B)** The body weight of mice bearing H292-Gef xenografts was measured on Days 0 and 7 and then once every three or four days (*n* = 5 per group). The error bars represent the means ± SD.

Supplementary Table 1: Sequences of the target gene-specific primers used in real-time PCR

Target genes		Sequences
AXL	Sense	5′-CGT AAC CTC CAC CTG GTC TC-3′
	Antisense	5′-TCC CAT CGT CTG ACA GCA-3′
PSEN1	Sense	5′-CCT CAA CAA TGG TGT GGT TG-3′
	Antisense	5′-TTG TGA CTC CCT TTC TGT GCT-3′
PSEN2	Sense	5′-CCG GGA TTC AGA CCT CTC T-3′
	Antisense	5′-GGC CAT GAA TGT GAG CAT AG-3′
ADAM10	Sense	5′-ATA TTA CGG AAC ACG AGA AGC TG-3′
	Antisense	5′-TCA ATC GCT TTA ACA TGA CTG G-3′
ADAM17	Sense	5′-CCT TTC TGC GAG AGG GAA C-3′
	Antisense	5′-CAC CTT GCA GGA GTT GTC AGT-3′
β-Actin	Sense	5′-AGC ACA ATG AAG ATC AAG AT-3′
	Antisense	5′-TGT AAC GCA ACT AAG TCA TA-3′
